# Comparison of pre and post Foley catheter Bishop’s Score: A retrospective record review at Aga Khan University Hospital Karachi, Pakistan

**DOI:** 10.12669/pjms.40.1.7433

**Published:** 2024

**Authors:** Ayesha Malik, Sheikh Irfan, Farheen Yousuf, Azra Amerjee, Sumaira Naz, Safna Virji

**Affiliations:** 1 Ayesha Malik, MRCOG, FCPS Assistant Professor, Department of Obstetrics and Gynaecology, Aga Khan University, Karachi, Pakistan; 2 Sheikh Irfan, MBBS, MPH,MHP,PhD Assistant Professor, Department of Obstetrics and Gynaecology, Aga Khan University, Karachi, Pakistan; 3 Farheen Yousuf, FRCOG, MCPS(HPE), FCPS Assistant Professor, Department of Obstetrics and Gynaecology, Aga Khan University, Karachi, Pakistan; 4 Azra Amerjee, FCPS Assistant Professor, Department of Obstetrics and Gynaecology, Aga Khan University, Karachi, Pakistan; 5 Sumaira Naz, FCPS Senior Instructor, Department of Obstetrics and Gynaecology, Aga Khan University, Karachi, Pakistan; 6 Safna Virji Resident, Department of General Surgery, Aga Khan University, Karachi, Pakistan

**Keywords:** Foley’s catheter, induction, Labor, prostaglandin E2, cervical ripening, Bishop score

## Abstract

**Objective:**

To compare pre and post Foley’s catheter Bishop Score during labour induction.

**Methods:**

This study was a retrospective study conducted at the Aga Khan University Hospital Karachi, Pakistan after approval from ethical review board. All women who underwent induction of labour with Foley’s Catheter at gestation of 37 weeks or more from September 2014-October 2015 were included. Data was entered and analyzed in Statistical Package for Social Sciences (SPSS) version 19.0. The comparison between pre and post Foley’s catheter Bishop Score during labour induction will be calculated by Wilcoxon sign test.

**Results:**

There were 981 cases of inductions of labour, 749 (76.3%) received Foley’s catheter, in combination with prostaglandins and oxytocin. About 68% were vaginal deliveries while 32% underwent C-section. Two third of women had bishop <4. Overall, Bishop score improved significantly in all patients with the catheter however, maximum benefit was seen in patients where the catheter was placed for 10-12 hours.

**Conclusion:**

Foley’s is the better and safer option. In view of our results, It has been recommended to keep the Foley’s for 10-12 hours to get significant improvement in bishop score.

## INTRODUCTION

Induction of labour is performed in 30% of pregnancies in UK.^1^ Its success depends on the state of cervix. The Bishop Score is the validated score, including cervical length, dilatation, position, consistency, and station of fetal head. If the score is less than 6-7, it is called as an unfavorable cervix.^2^,^3^ The recommended method of induction in these cases would be Dinoprost, misoprostol (pharmacological) or mechanical method.^2^

Dinoprost is recommended as drug of choice, however WHO recommends Misoprostol and Foley’s catheter as cost effective methods of induction. Both Dinoprost and Misoprostol carries risk of hyperstimulation by two to seven folds^2^. Hence WHO recommends mandatory availability of equipment and health personnel for fetomaternal monitoring.^2^,^4^

Recently mechanical induction with Foley’s or osmotic cervical dilators drew the attention of researchers and policy makers.^5^ A Meta-analysis just published compared osmotic dilator versus Dinoprostone concluded comparable cervical ripening with minimum risk of adverse effects and hyperstimulation.^6^ In 2022 RCOG affirmed, that mechanical method of induction has same C-section rates with delivery in 24 hours. The safety profile is substantial, reduces hyperstimulation with fetal heart abnormalities by 65% and decrease adverse neonatal outcome by 52%.^7^

The mechanical induction can be started with assessment of cervix and fetal cardiotocography (CTG). Next assessment is required either 12 hours after insertion of Foley’s or commencement of uterine contraction whichever is earlier. Low cost, high safety with minimum monitoring are the benefits that can make it as most favourable alternative in resource constraint facilities.^4^,^8^ This is also considered as the best option for Induction of labour at home or in cases with previous one C-section.^4^,^9^

Most of the literature available for induction of labour took delivery within 24 hours as the outcome^9^. In all these methods amniotomy and oxytocin are used in combination. Nevertheless, the sole effect of Foley’s catheter on bishop score is rarely studied.^10^,^11^

The DILAFOL controlled trail also proved that both Cervical osmotic dilator and Foley’s catheter are equally effective^5^. With regard to maternal satisfaction and pain score, the women felt more in control, mobile, easy to insert with lowest pain experience with mechanical method. During antenatal visits, the health professional must provide information leaflets.^2^,^6^,^7^. A recent Cochrane review further endorsed the use of Foley’s as one of the safest choice.^12^

This study focused mainly on Bishop score, aimed to compare the pre-and post-Foley’s catheter Bishop Score during labour induction and to assess the most effective and benefical time to keep it. The results of this study will give significant insight to develop guideline locally as well as in other low middle income countries (LMIC) to use as one of the safest and cost effective method of IOL.

## METHODS

This was a retrospective record review of all women who underwent induction of labour ≥37 weeks of pregnancy between September 2014 - October 2015, While women who were <37 weeks, Pre-labour rupture of membrane, previous cesarean section and in which insertion of intracervical Foley’s was not possible, were excluded. The duration of Foley’s catheter in hours was the dependent variable , while pre and post Foleys Bishop score was taken as an outcome variable. The initial evaluation includes a complete review of the antenatal record with maternal and fetal assessment and Bishop score.

This is our institutional policy to admit patients in the evening. The Foley’s catheter is to be placed between 18:00 hours to 22:00 hours in the ward after assessment of bishop score and satisfactory cardiotocography. The balloon was inflated with 50ml of fluid and taped with maternal thigh without any traction. They are shifted to the labor room the next morning around 7:00 am. The Enema is given followed by bath and removal of Foley’s with a reassessment of bishop score, followed by either prostaglandin or artificial rupture of membrane and augmentation with oxytocin according to bishop score and uterine contractions.

### Statistical Analysis

Data was entered and analyzed in Statistical Package for Social Sciences (SPSS) version 19.0. Mean and Standard deviation was calculated for continuous variables such as maternal age, gestational age at delivery and BMI at booking, parity and delivery. The categorical variables like method of induction, mode of delivery were taken in numbers and percentages. The comparison between pre and post Foley’s catheter Bishop Score during labour induction was calculated by Wilcoxon sign test. For pre and post categorical comparison, McNemar-Bowker Test or Marginal Homogeneity Test was applied. P-value of <0.05 is considered statistically significant.

### Ethical Approal

Permission from the ethical review board of the institution was taken with reference Number (3877-Obs-ERC-15). The information that was taken from medical records remained confidential. The patient’s name and identity were not disclosed at any time.

## RESULTS

A total of 981 women who underwent induction of labour were included in this study. The average age of the participants was 28.94±4.12 years. Almost 57% of the women were nulliparous. Foley’s in combination with prostaglandin, oxytocin, and (Artificial rupture of membranes)ARM was used in 76.3% women and only Foley’s catheter for labour induction was used in 23.6% women. Post-dates pregnancy (40+weeks) was the commonest cause of induction in 31.6% of the participants, while the medical reason for induction was found in 31.3%, its further breakup was 16.2% had Diabetes mellitus, 12.6% had hypertension /preeclampsia, growth restriction was found in 9.7% pregnancies. Ninety-five (9.2%) women had a social reason for induction.Demographic and induction characteristics of patients are shown in Table-I.

**Table-I:** Demographics and induction characteristics (n=981).

Variables	Statistics
Age (Years)	28.94 ± 4.12
BMI at booking (kg/m^2^)	26.490 ± 6.27
BMI at delivery (kg/m^2^)	30.125 ± 4.19
Gestational age at booking (Weeks)	14.29 ± 7.05
Gestational age at delivery (Weeks)	38.70 ± 1.66
***Parity***	
0	555(56.6%)
1	248(25.3%)
≥2	178(18.1%)
***Method of Induction***	
Foleys	232(23.6%)
Combined	749(76.3%)
***Indications for Induction***	
Post-dates(40+weeks)	310(31.6%)
Medical problems	308(31.3%)
SGA/IUGR baby	95(9.7%)
Social	90(9.2%)
Others	178(18.2%)
***Duration of Foleys***	
6-8 Hours	140 (14.3)
8-10 Hours	560 (57.1)
10-12 Hours	213 (21.7)
> 12 Hours	68 (6.9)
***Mode of delivery***	
SVD	591(60.2%)
Instrumental	72(7.4%)
LSCS	318 (32.4%)
Duration of foleys (hh:min)	9:30±2:00
Insertion to delivery time (hh:min)	17:00±6:25

Out of 981 women, most were delivered by spontaneous vaginal delivery (SVD) (60.2%). Lower segment caesarean section (LSCS) was performed in 32.4% (n=318) patients, and 7.3% (n=72) underwent assisted vaginal delivery. The most common indication of LSCS was non progress of labour, which was observed in 48.4% (154/318) of cases. Fetal distress was the second most common cause of Cesarean section accounting for 25.2% (80/318) of all deliveries.

The overall median pre and post bishop score was 4[IQR=2 & Range: 1-8] and 5[IQR=1; Range: 2-11]. Success rate of IOL was 67.58% (663/981) [95%CI: 64.55% to 70.51%]. The pre and post Foley’s Bishop score with all individual components were compared in Table-II. A statistically significant improvement was found between pre and post Foley’s bishop score, length of the cervix, cervical dilatation, consistency, and cervical position with pre and post Foley’s (p≤0.0005) respectively.

**Table-II:** Comparision of pre and post Foley’s

Variables	Pre Foleys	Post Foleys	P-Value
***Bishop Score (n=980)***			
<2	3(0.3%)	0(0%)	<0.0005†
2-4	666(67.9%)	154 (15.7%)	
5-6	288(29.4%)	693 (70.6%)	
>6	23(2.3%)	134 (13.7%)	
***Length of cervix (n=980)***			
>4 cm	3 (0.3%)	1 (0.1%)	<0.0005‡
3-4 cm	736 (75.0%)	515 (52.5%)	
1-2 cm	241 (24.6%)	465 (47.4%)	
***Dilatation (N=980)***			
Close	45 (4.6%)	2 (0.2%)	<0.0005‡
1-2	899 (91.6%)	533 (54.4%)	
3-4	35 (3.6%)	428 (43.6%)	
5	1 (0.1%)	17 (1.7%)	
***Consistency (n=966)***			
Firm	86 (8.9%)	4 (0.4%)	<0.0005‡
Medium	479 (49.6%)	207 (21.1%)	
Soft	401 (41.5%)	755 (77.3%)	
***Position (n=966)***			
Posterior	513 (53.1%)	275 (28.5%)	<0.0005‡
Midline	449 (46.5%)	82 (70.6%)	
Anterior	4 (0.4%)	9 (0.9%)	
***Station (n=979)***			
-3	969 (99.0%)	966 (98.7%)	0.225†
-2	10 (1.0%)	11 (1.1%)	
-1/0	0(0%)	2 (0.2%)	

‡McNemar-Bowker Test †Marginal Homogeneity Test, Pair-wise missing data were not include in analysis for each factors therefore total number of cases were not 981.

**Fig.1:**
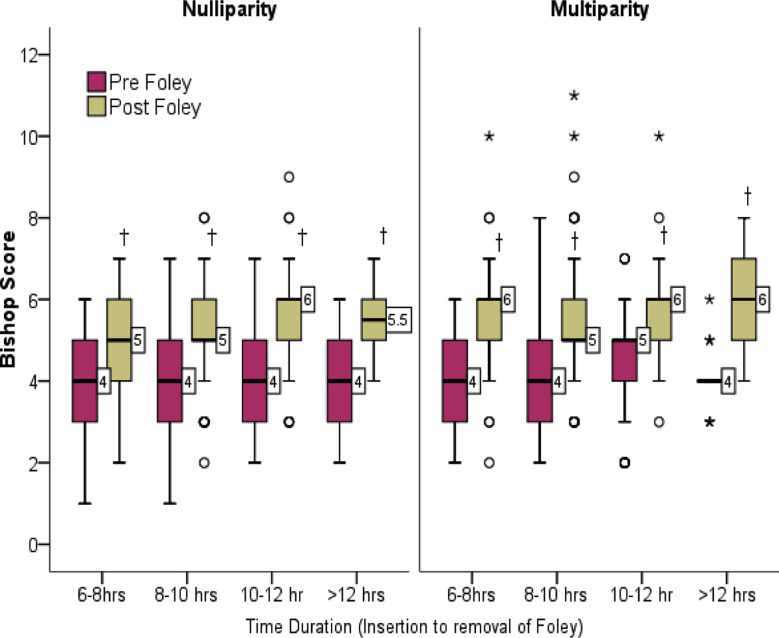
Comaprion of pre and post bishop score according to time duration of foley with successus of IOL stratified by parity. **†** Significant at p<0.05 * Extreme value and **o =**outliers.

The correlation between the duration of Foley’s catheter with the number of patients, and with pre and post-Foley’s bishop score (p-value=<0.005) respectively is shown in Table-III. Pre-Foley’s bishop median score within six-eight hours was four and 25^th^-75^th^ percentile was (three-five), simultaneously, the post-Foley’s bishop median score was 5 and 25^th^-75^th^ percentile was (five-six), followed by (eight-ten hours) pre-Foley’s bishop median 4 percentiles (three-five), post-Foley bishop median five percentile (5-6), (10-12 Hours) pre-Foley’s bishop [median 4 percentile (3-5), post-Foley’s bishop [median 6 percentile (five-six), and (>12 hours) pre-Foley’s bishop median four percentiles (3-4), and post- Foley’s bishop median six percentile (five-six). The pre and post Foley’s median bishop score among primigravida and multigravida patients, and the change in the median pre and post Foley’s scores were similar in both groups is shown in Fig-1.

**Table-III:** Comparison of pre and post median bishop score.

Duration of Foley Catheter	n	Pre-Foley Bishop Score Median [25^th^-75^th^ Percentile]	Post-Foley Bishop Score Median [25^th^-75^th^ Percentile]	P-Value*
6-8 Hours	140	4 (4-5)	6(5-6)	<0.005
8-10 Hours	560	4 (3-5)	5(5-6)	<0.005
10-12 Hours	213	4 (4-5)	6 (5-6)	<0.005
>12 Hours	68	4 (4-4)	6 (5-6)	<0.005

## DISCUSSION

This study was aimed to compare the pre and post Foley’s bishop score during labor induction. We found statistically significant improvement of Bishop score as a whole, along with all individual components were also improved significantly after use of intracervical Foley’s. This was further endorsed by RCT that showed a mean improvement in bishop score from 3.3 to 5.3 by Foley’s.^13^ A meta-analysis also confirmed that Bishop score is improved by 86% in the Foley’s group within 12 hours of insertion.^14^

In this study 56% of patients were primigravida, this is consistent with the study conducted in Civil Hospital Karachi.^15^ There were 68% vaginal deliveries and 32% C-section. The C-section rate was slightly higher in our data set in comparison to the study conducted in our secondary hospital at Karimabad with 18%. This could be due to the tertiary setup with higher complexity of cases at the main Aga Khan Hospital. It was further noted that women with a bishop score < 5 were 1.9 times more at odd of having a C-section.^16^

Our data set compared the bishop score in relation to different time ranges. We found that the vaginal delivery rate was highest in the group of 8-10 and 10-12 hours. The bishop score was progressed from 4/10 to 5/10 in 8-10 hours while in 10-12 hours the improvement in bishop score was from 4/ 10 to 6/10. However, there was no additional benefit of keeping the Foley’s for more than 12 hours. This had been affirmed by a randomized controlled trial that the delivery within 24 hours is maximum in the group where they kept the Foley’s for 12 hours, combined method of Foley’s with single dose of Dinoprost and augmentation with oxytocin leads to significant improvement in Bishop score (6.67vs 5.98 p=0.45) along with insertion to delivery interval of mean of 16 hours and 16 minutes.^11^ On the contrary, another RCT states that Foley’s for six hours leads to shorter insertion to delivery time in contrast to 12 hours.^17^

We used 50ml of fluid to inflate the balloon in all patients due to our institutional policy. An open labelled RCT which used a different amount of fluid from 30 ml-80ml didn’t find any difference with reference to vaginal delivery within 24 hours.^18^

When Foley’s alone was compared with prostaglandins, the labour duration remarkably increased. Foley’s catheter acts as a mechanical dilator of the cervix that locally releases the prostaglandins that only ripen the cervix without inducing uterine contractions. If it is combined with Prostaglandins or Oxytocin, it leads to a shorter induction to delivery by 2.7 hours (95% CI - 4.33 to-1.08, *p* = 0.001).^19^,^20^ A recent study compared Foley’s with PGE2 (prostaglandin E2) vs PGE2 depict statistically significant vaginal deliveries with shorter duration of labour in combined arm.^21^ Last year research conducted in Pakistan compared Foley’s with PGE2, failed to find any difference in labour duration and rate of C-section. However, there was significant cost difference with Foley’s catheter was 256 PKR while PGE2 1500 PKR.^22^

Due to its availability, cost-effectiveness, less monitoring, safety profile with an equal rate of C-section made mechanical induction as first and preferred choice.^7^ In future the more research can be considered to use Foley’s in out patient setting that will futher reduce the burden on the resource constraints health system in Pakistan.

### Limitations

It is a retrospective study with some missing data. However it was less than 10% , hence we are assuming that it will not significantly affect the results.Another limitation is the unequal number of patients in different groups of duration of intracervical Foley’s.There is a need in future for prospective study with randomized control design to prove and generalize the results in our population.

## CONCLUSION

Foley’s is the safer option for Low middle income countries. However, in view of our results, it has been recommended to keep the Foley’s for 10-12 to get significant improvement in bishop score.

### Authors Contributions:

**AM, FY**: Conceptualized and designed the study, and drafted the manuscript. They also assisted in data collection and shared in statistical design and analysis.

**IS:** Did data analysis and interpretation.

**AA, SN:** Participated in the study planning and assisted in data collection and entry.

**SV:** Was medical student and assisted in data collection.

All authors were responsible for the overall accuracy of this project contributed to the final review of the manuscript accuracy of this project contributed to the final review of the manuscript.
